# Control of Pain After Surgical Debridement of Burn Wounds

**DOI:** 10.5812/atr.6612

**Published:** 2012-08-21

**Authors:** Cheng Jie Zheng, Albert Y. Wu

**Affiliations:** 1 Department of Ophthalmology, Mount Sinai School of Medicine, New York, USA

**Keywords:** Burns, Debridement, Pain

***Dear Editor,***

We read with interest your article on the use of gabapentin pain control after burn debridement (1). Gabapentin has a well-established role in the management of neuropathic pain, and has been investigated in the past decade regarding its ability to potentiate the effect of morphine on postoperative pain, or decrease the amount of pain experienced by patients after surgery. This study represents one of the only randomized, placebo-controlled, double-blind studies to look at gabapentin in burn patients. The authors found that 1200mg of gabapentin 2 hours prior to operative debridement of burn wounds led to a significant decrease in pain in the immediate 24-hour postoperative period, as measured by the visual analog score and by patient-requested morphine consumption. While the results are important and consistent with the growing body of literature, there remain methodological questions that are not addressed (2-4).

First, while the authors state that total burn surface area in all patients were comparable (average of 13% ± 25 in the placebo group versus 19% ± 29 in the gabapentin group), there are several additional characteristics that can significantly affect pain, both pre-and post-operatively. Burn severity, type of burn (flame, chemical) and location on the body are not addressed here. In addition, the extent of debridement must also be considered, with regard to both surface area and depth. Conditions such as infection or other complicating medical conditions are also not mentioned in the article, which can be common in burn patients, and which can significantly affect patient outcome. Secondly, neuropathic pain induced by burns can lead to chronic and debilitating pain; this should be evaluated, depending on the feasibility of follow-up. The authors state that “Fassoulaki *et al.* (5) were unable to demonstrate a decrease in analgesic consumption and VAS scores at rest or after movement during the first 24h after the operation” due to “differences in the types of surgeries performed.” In fact, however, Fassoulaki *et al.* (5) were able to find that gabapentin reduced requirement for analgesics and led to decreased pain with movement within the first week post-operatively; the caveat was that this difference did not persist long-term (at 10 days or 3 months). Because chronic pain control can be such an important aspect of clinical outcomes in burn patients, this is critical to consider when thinking about impact on changes in management.

In summary, this study provides important support for the use of gabapentin in postoperative pain, and represents one of the only studies to address its use in burn patients. However, several questions regarding the clinical condition of patients (burn severity, type of burn, location of burn, extent of debridement, and presence of infection) must be considered; long-term sequelae with regard to chronic pain must also be addressed. All of these variables must be taken into account before the true impact of gabapentin in these patients can be determined.
